# Value of contrast-enhanced ultrasound in the differential diagnosis of ureteral tuberculosis from ureteral malignant tumour in women

**DOI:** 10.1017/S0950268823000419

**Published:** 2023-03-15

**Authors:** Wenzhi Zhang, Gaoyi Yang, Jianping Xu, Dali Wang, Tu Ni

**Affiliations:** Department of Ultrasonography, Affiliated Hangzhou Chest Hospital, Zhejiang University School of Medicine (Integrated Chinese and Western Hospital of Zhejiang Province, Hangzhou Red Cross Hospital), Hangzhou, Zhejiang, China

**Keywords:** Contrast-enhanced ultrasound, malignant tumour, tuberculous, ureter

## Abstract

This study aimed to evaluate the contrast-enhanced ultrasound (CEUS) features of ureteral tuberculosis (UTB) and ureteral malignant tumour and to explore its application value in the differentiation of UTB from ureteral tumour. The ultrasound (US) and CEUS imaging features of 33 and 12 cases of pathologically confirmed UTB and ureteral malignant tumour, respectively, were retrospectively evaluated, and echo of the ureteral wall, abnormal echo of the lumen, degree of ureteral dilation and CEUS patterns of the two diseases were statistically analysed. The results revealed that the lumen echo of UTB was hyperechoic or anechoic, whereas that of ureteral tumour lesions was hypoechoic (*χ*^2^ = 28.22, *P* < 0.001). The wall echo of the obstruction site differed between the two diseases; in UTB, the ureteral wall was thickened but the outer wall remained intact, whereas in ureteral tumour, both the malignant tumour wall and outer wall were irregular (*χ*^2^ = 30.25, *P* < 0.001). CEUS of UTB revealed nonenhancement or heterogeneous enhancement in the lumen, whereas that of ureteral tumours revealed significant homogeneous enhancement (*χ*^2^ = 30.25, *P* < 0.001). Thus, CEUS can reveal lesion microcirculation and be used to evaluate blood supply characteristics in the lesion, indicating that it has high potential for differentiating the two diseases.

## Introduction

Genitourinary tuberculosis accounts for 15% of all extrapulmonary tuberculosis cases and 3–4% of all tuberculosis cases [1], urogenital tract tuberculosis is the second to third most common form of extrapulmonary tuberculosis [2, 3]. The lack of highly sensitive tests, the emergence of drug-resistant strains of TB, and HIV co-infection are major factors contributing to inadequate global TB control [4]. Many women suffer from ureteral tuberculosis (UTB), which is an infectious disease and has malignant features on imaging. The differential diagnosis is often difficult, and the treatment options are completely different. Enhanced computed tomography or enhanced magnetic resonance imaging is often performed. Most patients with UTB and ureteral malignancy manifest impaired renal function. Therefore, contrast-enhanced ultrasound (CEUS) is increasingly used in the differential diagnosis of ureteral space-occupying lesions. This study aimed to evaluate the CEUS and conventional ultrasound (US) features of UTB and ureteral malignancy and summarise the value of CEUS in the differential diagnosis of the two diseases.

## Methods

### Patients and study design

This study was reviewed and approved by the Medical Ethics Committee of Affiliated Hangzhou Chest Hospital of Zhejiang University, and all patients provided informed consent. A total of 30 UTB cases and 12 ureteral malignant tumour cases confirmed via pathology in Hangzhou Red Cross Hospital from March 2015 to April 2022 were examined using US and CEUS. The ureteral lesions of the patients were surgically excised to obtain pathological data. The inclusion criteria were as follows: (1) patients who underwent US and CEUS and had complete medical information and (2) patients diagnosed with ureteral tumour and UTB who demonstrated positive features in the ureteral wall or lumen via US and CEUS. The exclusion criteria were as follows: (1) patients with double ureteral malformation on the affected side, (2) patients with small ureteral tumours that cannot be demonstrated via US, (3) patients with a history of severe allergy who are not suitable for CEUS and (4) patients with severe cardiopulmonary dysfunction that does not permit the use of CEUS.

### US and CEUS examination

A Philips ultrasonic diagnostic instrument (iu22, Philips Healthcare, Bothell), high-frequency linear array probe (L12-5, frequency 5–12 MHz; L9-3, frequency 3–9 MHz) and convex array probe (C5-1, frequency 1–5 MHz) were used for patient examinations.

During the examination, the patients assumed a prone, lateral or recumbent position, with their abdomen and waist fully exposed, and the scanning range was from the kidney to the upper, middle and lower segments of the ureter and bladder. The entire process was recorded, and scanning was performed gradually. Lesions in the ureter lumen and ureter wall were evaluated using US and CEUS, and the ureter diameter was measured (at the widest part of the ureter); a routine examination of the contralateral ureter was also performed. The following findings were recorded: location of the lesion, the echo of the lesion and the ureteral wall, any calcifications, and the internal colour blood flow signal.

SonoVue (Bracco SpA, Milan, Italy) was used as the US contrast agent. Its main component is second-generation sulphur hexafluoride microbubbles, which are approved for diagnostic US imaging in China, and all patients provided informed consent prior to CEUS examination. The instrument was adjusted to low mechanical index (0.06) pulse reverse harmonic imaging during CEUS. A rapid injection of 2.4 ml of contrast agent was administered through the elbow vein, followed by 5 ml of saline solution. Ureteral lesions were scanned immediately after injection and recorded continuously for approximately 120 s, and the images were saved.

To reduce subjective error, both routine US and CEUS enhancements were performed by two attending physicians with 5 years of experience in US diagnosis, who were blinded to the pathological results. The data were independently diagnosed and analysed by two sonographers, followed by a discussion to unify the results.

### Statistical analysis

Data were analysed using SPSS 23.0 (IBM Corp., Armonk, NY, USA). Count data for the difference between routine US and CEUS of the ureteral lesions of different pathological types were analysed using the *χ*^2^ test and Fisher's exact test. A *P* value of <0.05 was considered statistically significant.

## Results

### Patients and study design

Both the UTB group (33 cases) and ureteral tumour group (12 cases) consisted entirely of women who were aged 18–68 and 53–82 years (mean age, 36.58 ± 7.46 and 61.34 ± 4.65 years), respectively. Table 1 shows the clinical symptoms and lesion sites of the patients. All the UTB and ureteral tumour cases were pathologically confirmed via ureteroscopy and cystoscopy biopsy. Among them, six cases of primary ureteral tumours (transitional cell carcinoma) underwent surgical treatment. The obstruction site was the upper ureter in two cases, the middle ureter in one case, and the lower ureter in three cases. Six cases of metastatic carcinoma with obstruction in the lower ureter were treated conservatively, and the primary focus was cervical cancer in five cases and rectal cancer in one case.

**Table 1.** General situation of patients in the two groups

Information	TB (*N* = 33)	Malignancy (*N* = 12)
Mean age (years)	36.58 ± 7.46	61.34 ± 4.65
Side (*N*%)		
Unilateral	25	8
Bilateral	8	4
Lesion in the ureter (*N*%)		
Upper segment	7	2
Middle segment	0	1
Lower segment	5	9
Entire segment	20	0
Symptoms (*N*%)		
Pyuria	4	0
Haematuria	0	9
Abdominal pain	12	2
Not specific	17	3

TB, tuberculosis.

### US examination

**Fig. 1.**
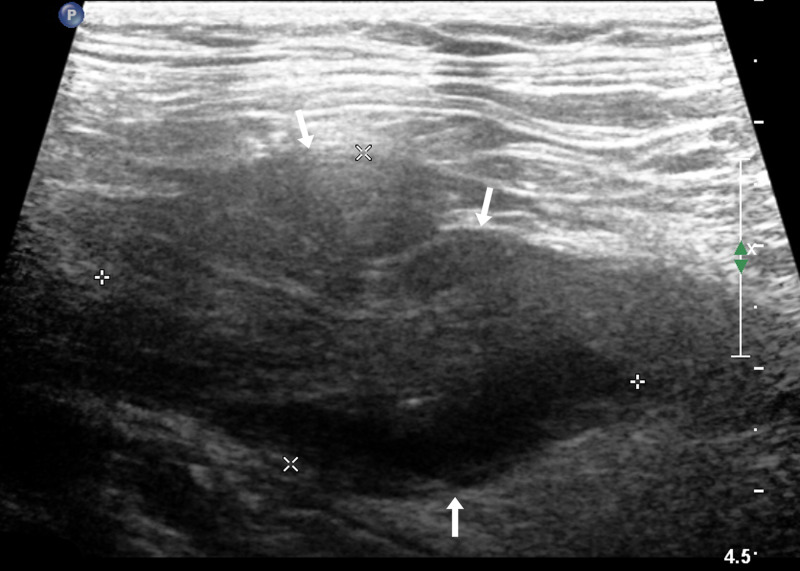
An 82-year-old woman with a malignant tumour in the upper ureter, which was hypoechoic in the ureter lumen with an irregular external wall (arrow).

Routine US showed that 25 cases of UTB and 8 cases of ureteral malignant tumours were single lesions, and the remaining cases were multiple lesions. Ureteral malignant tumours were mostly characterised by low echo in the lumen and thickened or irregular wall at the obstruction site (Fig. 1), which was irregular in 75.0% (9/12) of the study participants.

**Fig. 2.**
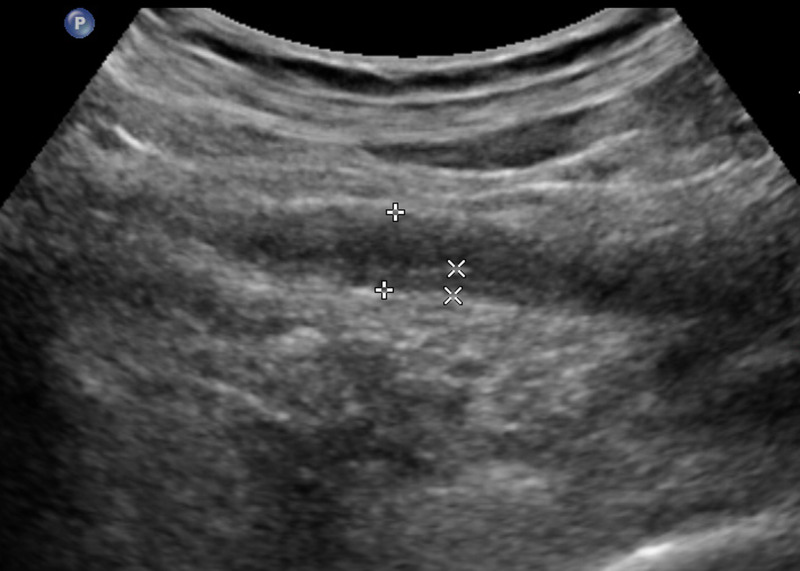
A 36-year-old woman presented with right ureteral tuberculosis with a widely thickened wall and extensively narrowed lumen with a regular external wall.

In the UTB group, the ultrasound features were diverse, with a high echo in the ureteral lumen, followed by no echo and low echo. Compared with the ureteral tumour group, the ureter wall was thickened widely and the mucosa was smoother in the UTB group (Fig. 2). The external ureteral wall of the UTB group was mostly complete and smooth, with calcification foci in 4% of the ureteral wall. Table 2 shows the internal echo of the ureter lumen; echo, smoothness and calcification of the ureter wall; and dilation of the ureters.

**Table 2.** Comparison of UTB and ureteral malignant tumour with routine ultrasound

	TB (*N* = 33)	Malignancy (*N* = 12)	*χ*^2^	*P*
Mean inner diameter of dilated ureteral lumen	0.78 ± 0.93	1.2 ± 0.64		
Echogenicity in the lumen in US			28.22	<0.001
Hyperechogenicity	16	0		
Hypoechoic	4	12		
Anechoic	13	0		
CDFI of abnormal echo in the lumen				
Yes	0	8		
No	33	4		
Wall morphology of the obstruction site			11.37	<0.001
Thickened	31	6		
Atypical	2	6		
Ureteral wall calcification foci				
Yes	4	0		
No	0	0		
External wall of the ureter			30.25	<0.001
Irregular	0	9		
Regular	33	3		

UTB, ureteral tuberculosis; TB, tuberculosis; US; ultrasound; CDFI, Colour Doppler flow imaging.

### CEUS examination

**Fig. 3.**
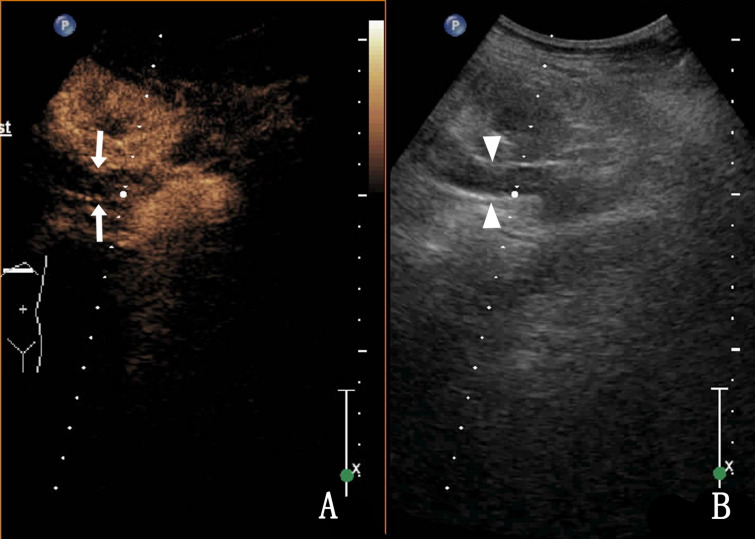
A 36-year-old woman with right ureteral tuberculosis. (a) nonenhancement in the lumen and (b) a hypoechoic upper ureter (the ureter is represented by triangular arrows).

Thirty-three cases of UTB showed heterogeneous enhancement in the lumen (18.2% [6/33]) and nonenhancement in the lumen (81.8% [27/33]) (Fig. 3), and most of them were low enhancement. The thickness of the tube wall showed homogeneous enhancement in 54.5% (18/33) and heterogeneous enhancement in 45.5% (15/33) of the cases.

**Fig. 4.**
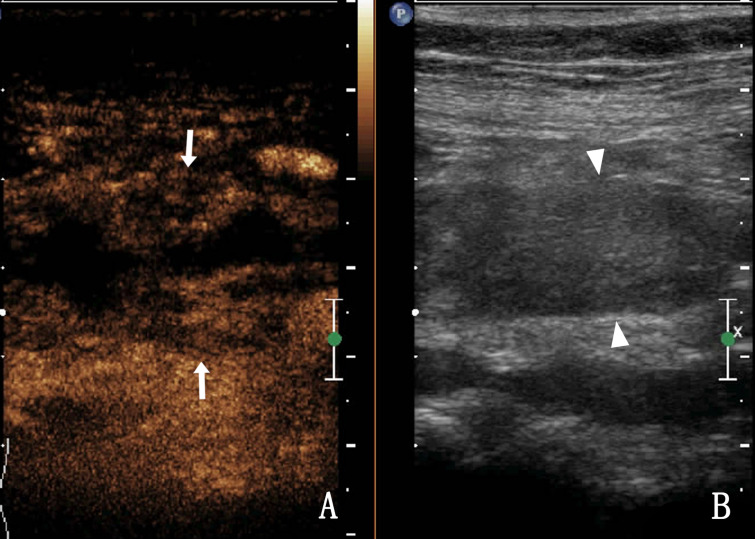
A 68-year-old woman with a right ureteral malignant tumour. (a) The enhanced area of the lesion after CEUS compared with 2D US (arrow); (b) The ureter lumen (triangle arrow) was hypoechoic in the 2D US.

Homogeneous enhancement was observed in 75.0% of the ureteral malignant tumours (9/12), heterogeneous enhancement in 25.0% (3/12), high enhancement in 66.7% (8/12) and low enhancement in 33.3% (4/12). After CEUS, there were five cases (41.7%, 5/12) with larger lesion areas compared with that observed in two-dimensional (2D) US (Fig. 4). Table 3 shows the CEUS features of the two groups. The coincidence rate of ultrasound and CEUS diagnosis and pathology were shown in Table 4. Compared with US, CEUS has statistical significance in the diagnosis of UTB and ureteral malignancy.

**Table 3.** Comparison of UTB and ureteral malignant tumour using CEUS

	TB (*N* = 33)	Malignancy (*N* = 12)	*χ*^2^	*P*
Ureteral lumen enhancement pattern			30.25	<0.001
Homogeneous enhancement	0	9		
Heterogeneous enhancement	6	3		
Nonenhancement	27	0		
Ureteral wall enhancement pattern			8.00	<0.005
Homogeneous enhancement	18	12		
Heterogeneous enhancement	15	0		
Intensity of enhancement				
High enhancement	3	8		
Low enhancement	30	4		
Enhanced area is larger than that of the 2D US			37.00	<0.001
Yes	0	5		
No	33	7		

UTB, ureteral tuberculosis; CEUS, contrast-enhanced ultrasound; US; ultrasound.

**Table 4.** The coincidence rate of ultrasound and contrast-enhanced ultrasound diagnosis and pathology (*n*)

Types of disease	US consistent with pathology	CEUS consistent with pathology	X2	*P*
Ureteral tuberculosis (30)	11	21	6.696	0.010
Ureteral malignancy (12)	7	12	6.316	0.012

## Discussion

Urinary tract infection is considered the most common urinary tract disease, affecting approximately 150 million individuals worldwide annually [5]. In UTB, the ureter and bladder are usually involved at a later stage after kidney involvement, as the tuberculous bacteria invade via a descending infection. UTB symptoms are nonspecific and include frequent urination, microscopic haematuria, lumbago, sterile pyuria, acidic urine and persistent cystitis [6]. Severe urinary tract infections can lead to kidney failure. Figueiredo *et al*. reported that UTB affects more men than women; the ratio of male to female was 2:1.15 to 2:1.3 [7]. The onset of UTB usually lags between 2 and 36 years (mean 22 years) behind the onset of initial TB, and a significant number of women are able to tolerate UTB. Biological culture or histopathology is the gold standard for a definitive diagnosis [8].

Primary ureteral malignancies are extremely rare, with transitional cell carcinoma accounting for 90% of all ureteral malignancies and squamous cell carcinoma and adenocarcinoma accounting for the remaining 10% [9–12]. Intake of arsenic-contaminated water, urinary stones, dialysis and Chinese herbal medicines are considered to be important causes of urothelial carcinoma [13, 14]. The occurrence of ureteral malignant neoplasms is uncommon and is associated with a poor prognosis owing to the difficulty in early detection. The most common symptom is haematuria or lumbago [15].

Accurate imaging diagnosis is the key to guiding treatments. However, the imaging findings of a ureteral malignant tumour may overlap with those of UTB, and both diseases often affect renal function [5, 16]. Therefore, simple, feasible and minimally damaging tests are needed to distinguish UTB from a ureteral malignant tumour. US image quality may be limited by factors such as the patient's body, intestinal gas interference and the US operator's skills. The influence of factors that cause an expansion in the ureteral lumen may render the tumour sample cloudy, which may lead to errors in the ultrasonic measurement and failure to determine the actual lesion size [17] or the edge or vascular signal may not be fully displayed [18]. In this study, the UTB group showed no colour blood flow signal in the ureteral wall and lumen, whereas the ureteral malignant tumour group showed approximately 66.7% (8/12) of blood flow. Moreover, US showed a relatively satisfactory rate of lesions in the lower and upper ureteral segments. In recent years, CEUS has been introduced as a new technology. In US imaging, contrast agents are used and administered into the blood vessels; this mainly evaluates blood perfusion in the organs, such as the lungs and liver. Therefore, it is considered a very safe examination and can be applied to many important organs of the body [19, 20], as corroborated in reports [17, 21]. The rate of CEUS in the upper urinary tract tumours was 94.4%–94.8%; thus, CEUS has great clinical potential in the differentiation of UTB and ureteral malignant tumours. In this study, compared with routine ultrasound, CEUS showed statistically significant difference in the diagnosis of UTB and ureteral tumour.

Ureteral malignancies occur primarily in the elderly, with a mean age of approximately 64 years at diagnosis [16]. In this study, the average age of the malignant tumour group was higher than that of the UTB group, and a malignant tumour should be excluded first for ureteral lesions in elderly patients. It has been suggested that 63% of ureteral renal cancers occur on the left side and 70% on the lower-third of the ureter [16, 21]. In this study, the incidence rate of unilateral ureteral malignant tumour was 66.7% (8/12) and that of bilateral malignant tumour was 33.3% (4/12), which may be related to the fact that six cases in this study were metastatic cancer rather than a primary ureteral malignant tumour. For example, bilateral ureteral dilatation and hydronephrosis are often detected in the late stage of cervical or rectal cancer. However, the degree of hydronephrosis in both diseases needs further study.

It has been reported that ultrasonographic findings of ureteral neoplasms are frequently hypoechoic in the intraluminal area in the following order: distal ureter, middle ureter and upper ureter [21, 22]. In this study, the 12 cases of ureteral malignant tumours were mostly located in the lower ureter, and the lower ureter is also a common site of UTB. However, UTB often involves the entire ureter. Therefore, patients with whole ureteral disease are more prone to UTB than those with local disease. In this study, 12 cases of ureteral malignant tumours were characterised as a low echo. The sonographic findings of UTB were diverse [8]; within the ureter, it was mainly hyperechoic, followed by anechoic and then hypoechoic, which may be attributed to the pathological process. Moreover, the main pathological changes of tuberculosis include effusion, caseation and granulomas. These three pathological changes often exist simultaneously. After thickening and stricture of the ureteral wall, necrotic deposits block the ureteral lumen, resulting in echo diversity.

The ureteral duct wall at the point of obstruction in the ureteral malignant tumour group was usually thickened or irregular [23], whereas that in the UTB group was thickened and had a wider and relatively smooth mucosal surface. The ureteral wall of the UTB group was usually complete and smooth, and 12.1% (4/33) of the ureter wall was calcified. Conversely, the ureter wall of the malignant tumour group was irregular, and 75.0% (9/12) of the ureter wall was irregular in this study.

The ureteral wall may be homogeneously enhanced in both diseases, and in CEUS, part of the ureteral wall shows heterogeneous enhancement in UTB. These wall changes may be caused by infection, irregular wall thickening, urothelial ulcer and caseation, followed by fibrosis and narrowing [24], and are usually located at the junction between the bladder, ureteral or pelvic ureter connection. The ureteral wall shows homogeneous enhancement. In the late stage, ureteral stenosis and obstruction are further caused by ureteral wall fibrosis, calcification, bladder volume reduction and bladder wall fibrosis. Moreover, necrotic material further accumulates in the ureteral lumen, forming hyperechoic or hypoechoic enhancements, whereas CEUS shows nonenhancement.

It has been reported that the CEUS of ureteral tumours shows homogeneous enhancement of lesions [17, 25]. In the 12 cases of ureteral malignant tumours in this study, 75.0% (9/12) showed homogeneous enhancement of CEUS, 25.0% (3/12) showed heterogeneous enhancement, 66.7% (8/12) showed high enhancement, and 33.3% (4/12) showed low enhancement. This finding may be related to the fact that some cases were metastatic tumours, and all three cases with heterogeneous enhancements were metastatic tumours.

In this study, 81.8% (27/33) of the UTB group showed nonenhancement of the abnormal echo during CEUS in the ureter, whereas 18.2% (6/33) showed heterogeneous enhancement. Furthermore, there were no cases with homogeneous enhancement in the ureter lumen. Additionally, in the malignant tumour group, there was no case of nonenhancement in the ureteral lumen. Therefore, the ureter was found to have an abnormal echo, which was considered UTB if there was nonenhancement and malignant tumour if there was a homogeneous enhancement.

The CEUS enhancement areas in the ureteral lesions were larger than those observed in 2D US, which was observed in 41.7% (5/12) of cases in the malignant tumour group. In contrast, in the UTB group, after CEUS, no changes in lesion size were observed. We believe that malignant tumours invading the ureteral wall and encompassing the surrounding fascia and organs lead to an enhanced volume increase. Thus, if a larger lesion area after CEUS is identified compared with 2D US, a malignant tumour should be considered.

The use of CEUS for the identification of heterogeneous enhancements in the ureteral lumen in UTB and ureteral malignant tumours poses certain difficulties. The findings need to be correlated with clinical symptoms and combined with history, laboratory examination of the urine and other imaging data to arrive at a comprehensive judgment. Further research is necessary for the differential diagnosis of heterogeneous enhancement of UTB during CEUS in the ureteral lumen, including ureteral malignant tumours.

## Conclusion

The limitation of this study is that only women patients were included. Moreover, the insufficient number of cases might have a certain impact on the reliability of the results. CEUS can reveal lesion microcirculation and be used to evaluate blood supply characteristics in the lesion. Two diseases are implicated when there is an abnormal echo in the ureteral lumen. When CEUS shows nonenhancement in the lumen and the ureteral wall is thickened and there is a heterogeneous enhancement, it suggests UTB, whereas a homogeneous enhancement of an abnormal echo in the lumen and a larger lesion area after CEUS compared with 2D US suggests a ureteral malignant tumour. Thus, CEUS is of great value in differentiating the two diseases. This study serves as a reference for female patients with ureteral obstruction in the future and provides direction for determining the cause of obstruction.

## Data Availability

The raw data supporting the conclusions of this article will be made available by the authors without undue reservation.
